# Editorial

**DOI:** 10.1120/jacmp.v2i1.2622

**Published:** 2001-01-01

It is with sorrow that we note the death of Jimmy Fenn. His obituary, written by James Purdy, follows this editorial. Jimmy was a personal friend for more than 30 years and he understood, better than most, what practicing clinical medical physicists faced in their daily work. He was one of them and he had a lifelong interest in their professional needs and clinical responsibilities. He wholeheartedly supported the ACMP and the formation of this journal, dedicated to the activities of clinical medical physicists. He will be missed. To his wife, family, and many friends we send our heartfelt condolences.

Most of you will have noted by now that we have a new Managing Editor, Richard Stark. Richard takes over for Anne Wright. The ACMP, this journal, and this editor will never be able to repay the debt we owe her. She was my choice for Managing Editor and for the past two years, during the formation of the JACMP, she worked tirelessly. Her help was invaluable. Thank you Anne we wish you well.

You will also note that we have some additional Associate Editors. Michael Herman has joined John Horton as an Associate Editor for Radiation Oncology Physics, and Ed Jackson has joined Stewart Bushong as an Associate Editor for Medical Imaging. As the journal grows we will continue to add new associate editors as the need arises.

Peter R. Almond, Ph.D.

Editor‐in‐Chief

